# Portable biosensor for monitoring cortisol in low-volume perspired human sweat

**DOI:** 10.1038/s41598-017-13684-7

**Published:** 2017-10-17

**Authors:** David Kinnamon, Ramesh Ghanta, Kai-Chun Lin, Sriram Muthukumar, Shalini Prasad

**Affiliations:** 1 Department of Bioengineering, University of Texas at Dallas, 800 West Campbell Road, Richardson, TX 75080 USA; 2 Enlisense LLC, 1813 Audubon Pond way, Allen, TX 75013 USA

## Abstract

A non-faradaic label-free cortisol biosensor was demonstrated using MoS_2_ nanosheets integrated into a nanoporous flexible electrode system. Low volume (1–5 μL) sensing was achieved through use of a novel sensor stack design comprised of vertically aligned metal electrodes confining semi-conductive MoS_2_ nanosheets. The MoS_2_ nanosheets were surface functionalized with cortisol antibodies towards developing an affinity biosensor specific to the physiological relevant range of cortisol (8.16 to 141.7 ng/mL) in perspired human sweat. Sensing was achieved by measuring impedance changes associated with cortisol binding along the MoS_2_ nanosheet interface using electrochemical impedance spectroscopy. The sensor demonstrated a dynamic range from 1–500 ng/mL with a limit of detection of 1 ng/mL. A specificity study was conducted using a metabolite expressed in human sweat, Ethyl Glucuronide. Continuous dosing studies were performed during which the sensor was able to discriminate between four cortisol concentration ranges (0.5, 5, 50, 500 ng/mL) for a 3+ hour duration. Translatability of the sensor was shown with a portable form factor device, demonstrating a comparable dynamic range and limit of detection for the sensor. The device demonstrated a R^2^ correlation value of 0.998 when comparing measurements to the reported impedance values of the benchtop instrumentation.

## Introduction

Wearable diagnostic biosensors for the detection and monitoring of analytes in transdermal sweat present an intriguing pathway toward improving user health outcomes in an inexpensive and noninvasive manner^1^. Transdermal biosensors have become a more viable option in recent years, due in large part to the miniaturization of conventional sensing mechanisms. Microneedle biosensors demonstrate encouraging results in the monitoring of glucose as well as select biomarkers in peripheral blood and interstitial fluid, but these devices are still minimally invasive as they puncture the skin in the order of microns, which in some cases can cause discomfort and infection to the user^2^. Current transdermal sweat biosensors require relatively large volumes (10–100 μL) in a localized region and count on chemically-induced sweating to attain sufficient sample volume^3^. Thus, there exists a challenge in designing wearable sweat-based sensors that can report and quantify biomarkers of interest from low volumes (i.e. 1–5 µL) of human sweat in a completely non-invasive yet still cost-effective manner. There are two major population sub-sets that stand to benefit from low volume sweat-based biosensors, with the first being the percentage of the population considered to be generally sedentary (i.e. sweat rate of 1–5 nL/min/gland). The Physical Activity Council reported in 2016 that 27.5% of the U.S. population was considered completely inactive or sedentary, and an additional 20.1% considered mostly inactive^4^. Physical inactivity plays a role in several chronic health conditions ranging from type-2 diabetes, stress, heart disease, and cancer^5^. Thus, there is a crucial need to monitor biomolecules associated with such chronic health conditions in a dynamic and non-invasive manner.

The second sub-set of the population is comprised of highly specialized cohorts, namely astronauts and future space tourists. Dynamic monitoring of specific biomarkers is essential in the diagnosis of fluctuating conditions within the human body which can result from prolonged states and/or exposures related to space travel, where conventional clinical diagnostics methods is still a developing field^6^. The degradation in the immunological response of astronauts due to space travel has been well documented, with findings making it increasingly clear that this decay is in large part due to the great physical and psychological stress an astronaut undergoes in preparation and execution of a space mission^7,8^. These factors include, but are not limited to: launching and landing gravitational forces, microgravity, radiation, malnutrition, disturbance in circadian rhythms, anxiety related to mission danger, confinement, and isolation from family and friends^8^.

The relationship between the physiological stress states listed above and cortisol levels in blood, saliva and sweat has been well established^9,10^. Cortisol is released in response to stress and low blood-glucose concentration. It functions to increase blood sugar through gluconeogenesis, suppress the immune system, and aid in the metabolism of fat, protein, and carbohydrates^11^ while also decreasing bone formation^12^. This allows for the prospect of using relative changes in cortisol expression as a means of monitoring a potentially immuno-compromised state^13–17^. In addition to being an indicator for the immune system, changes in cortisol expression can be used to diagnose Cushing’s syndrome and Addison’s disease, whose symptoms include: fatigue, falling blood pressure, irritability, poor concentration, and depression^18,19^. Thus, measurement of cortisol levels in the body can be an important diagnostic tool in everyday life, clinical settings, and during stress-intensive activities. Cortisol levels in the body fluctuate throughout the day with levels being highest in the morning and lowest in the evening. Dietary intake and metabolic imbalances cause further fluctuations. To detect relative deviations in the body’s natural fluctuations, a dynamic determination of cortisol levels is needed without the use of costly and cumbersome laboratory equipment.

There is currently a small selection of portable or point-of-care diagnostic tools for detection of cortisol that can be found in Table 1. Electroanalytical techniques are desired over other transduction methods due to increased accuracy and sensitivity with minimal instrumentation that utilize low power sources. The resulting electrochemical signal can be easily and reliably analyzed lending itself to a wearable form factor that is relevant for the presented applications. The use of portable electroanalytical techniques in sweat-based wearables ranging from amperometry, cyclic voltammetry, chronoamperometry, and electrochemical impedance spectroscopy (EIS) has been investigated with increased interest in recent years. Outside of our group, most efforts have been to monitor the general physiology of the user through measuring analytes such as sodium, zinc, ammonium, lactate, glucose, ethanol, or pH^3,20–22^. For this work, non-faradaic EIS was targeted for its ability as a label-free method for rapid and highly specific affinity-based detection of target molecules. This can be accomplished by measuring effective changes in the resistive and capacitive behaviors of the sensor due to specific binding events of the specific biomarker, in this case; cortisol onto a transduction element to quantify cortisol levels present in low volumes of human sweat.

**Table 1:** Comparison of various sensors for cortisol with respect to substrates, buffer media, linear range and detection limit.

Substrate	Detection method	Buffer media	Dynamic range	Detection limit	Ref.
Au nanowire	Square wave voltammetry	PBS	3.7–12 µg/mL	3.7 µg/mL	43
Immune-chromatographic strip	Immuno-chromatography	PBS	1–10 ng/mL	1 ng/mL	44
Disposable disc-chip (acrylic resin)	Immuno-chromatography	Saliva	0.4–11.3 ng/mL	0.4 ng/mL	45
SAM modified microfabricated interdigitated Au electrodes	Cyclic voltammetry	PBS	3.6 pg/mL –36 ng/mL	3.6 pg/mL	46
Zinc oxide on flexible nanoporous membrane	Electrochemical Impedance Spectroscopy	Human sweat	10–200 ng/mL	1 ng/mL	30
MoS_2_ Nanosheet on flexible nanoporous membrane	Electrochemical Impedance Spectroscopy	Human sweat	1–500 ng/mL	1 ng/mL	This work

Physiological relevant range reported for cortisol in human sweat is 8–140 ng/mL^10^.

Recently, layered two-dimensional (2D) inorganic transition-metal dichalcogenides (TMDs), primarily Molybdenum disulfide (MoS_2_), have been investigated for many applications^23,24^. In nanoscale morphology, 2D MoS_2_ edges and corners can be engineered with sulfur termination, giving the opportunity to do further surface chemical modification suitable for biosensing. Similar to other 2D materials, MoS_2_ offers increased surface areas that enhance its biosensing performance. Due to the semi-conducting property and appropriate bandgap of 2D MoS_2,_ where a direct bandgap of single-layer MoS_2_ is ~1.8 eV, the sensitivity of 2D MoS_2_ based devices is higher compared to other 2D materials^25–29^. The target molecules bind on the MoS_2_ altering the charges on the surface, a process which is typically reflected as a change to the measured capacitive reactance. The added charges on the surface also change the impedance, which can be detected by EIS.

**Figure 1:**
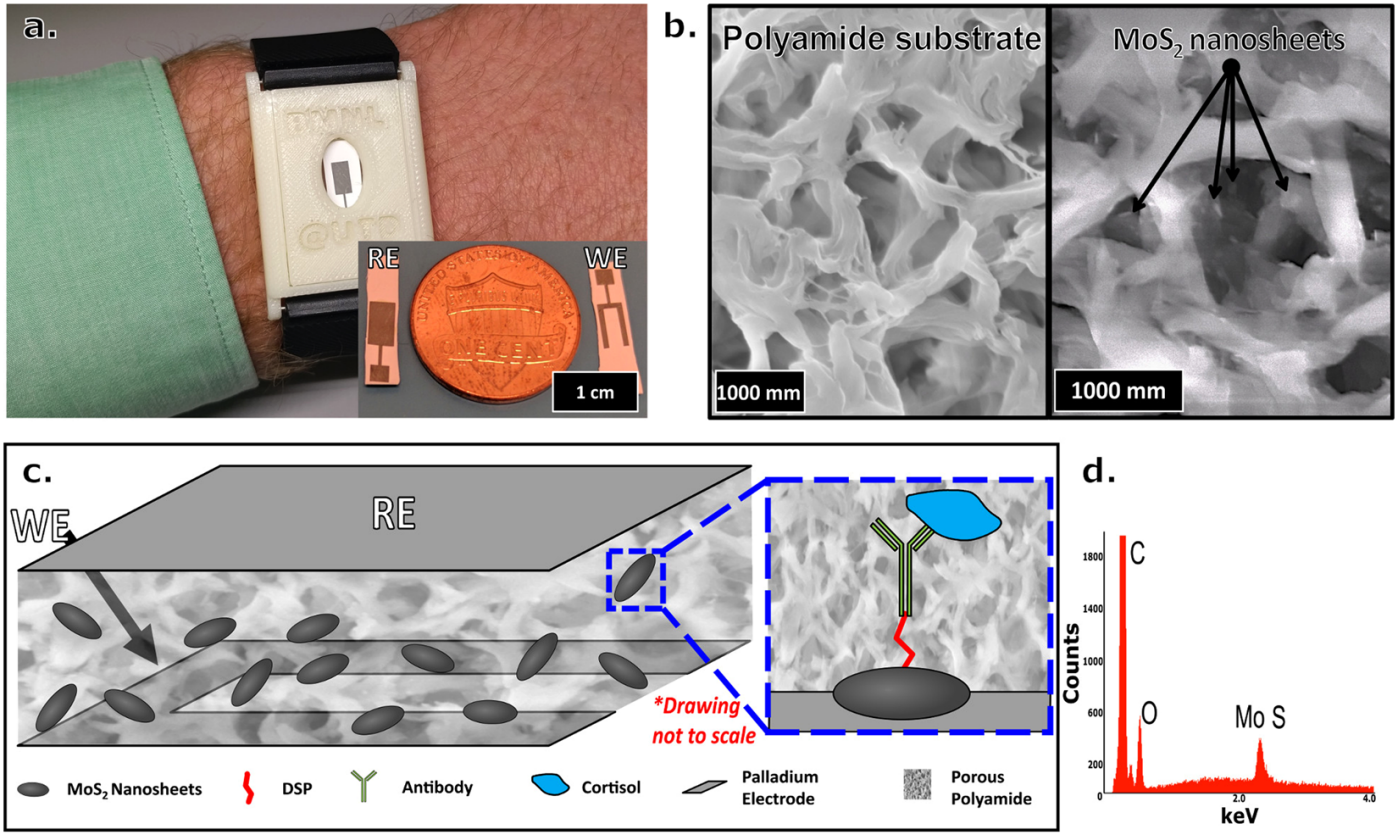
(**a**) Demonstration of envisioned device prototype form factor for transdermal monitoring of cortisol in human sweat. Inset: close-up of the sensor with a penny for reference. (**b**) SEM image of blank polyamide membrane (left) and MoS_2_ nanosheets deposited into porous polyamide membrane (right). (**c**) Schematic drawing of the MoS_2_-through membrane sensing platform for electrochemical affinity-based detection of cortisol. The picture depicts the nanosheets embedded between the reference and working electrode within the polyamide membrane. The blue box, zooms in on a single nanosheet and depicts the constructed affinity assay for cortisol. (**d**) EDAX spectra related to the highlighted zone in the SEM micrographs showing MoS_2_ is embedded in the membrane.

In this work, a novel low volume sensing platform that builds an affinity-based detection mechanism utilizing low-cost semi-conductive MoS_2_ nanosheets dispersed within the pores of a porous polyamide (PA) membrane has been demonstrated. The hydrophilicity of the PA membranes allows for low volume (<5 µL) diffusion driven total coverage of the sensing region which offers several benefits compared to conventional lateral flow assays in areas such as sample volume, assay completion time, and impact of microgravity on sample kinetics. This is critical for maintaining the electrode-electrolyte interface in highly diverse environments. The implementation of the dispersed MoS_2_ nanosheets between vertically aligned electrodes, when contrasted with a standard planar electrode system, allows for three-dimensional (3D) sensing where the transduced electroanalytical output is measured perpendicular to the direction of the applied inputs signal, allowing sensing through the membrane substrate as opposed to just the surface. MoS_2_ increases the sensitivity of the sensor even further to detect cortisol across physiologically relevant concentrations while providing a location for cross-linker binding^10^. Many detection schemes utilize rigid material platforms such as silicon in conjunction with MoS_2_, making the system difficult to translate to wearable technology. To our knowledge, this work and our previous cortisol work with ZnO are the only examples of non-faradaic and label-free cortisol sensing on a flexible substrate platform in human sweat^30^. The confined nature of the embedded MoS_2_ nanosheets combined with the flexible polyamide allows for direct contact of the polymer with regions of sweat-producing skin and allows for conformation to human body morphology^31^. Additionally, a portable electronic research prototype device was developed and tested alongside the standard lab instrument, demonstrating the translatability of this technology to a true portable and wearable form factor. A demonstration of the envisioned device form-factor is presented in Fig. 1a where the disposable sensor can non-invasively interface with the user’s skin.

The presented MoS_2_ functionalized biosensor demonstrates a limit of detection for cortisol of 1 ng/mL on both the lab potentiostat and in the portable electronic device. This range sufficiently covers the physiological expressed range of cortisol in human sweat reported between 8.16 to 141.7 ng/mL by Russell *et al*.^10^. Though sharing the same limit of detection (LOD), this sensor demonstrates a superior dynamic range of 1–500 ng/mL when compared to the 10–200 ng/mL dynamic range previously reported by our group in human sweat^30^. Our previous device showed time-point based detection whereas the current sensor technology, proposed here, demonstrates the ability to continuously detect cortisol for a duration of 3+ hours across the established sensor dynamic range with a 7-minute refresh rate. This work is the first demonstration of an electrochemical MoS_2_ biosensor for the quantification of cortisol in ultra-low (1–5 μL) volumes of human sweat, utilizing a vertically aligned electrode system towards the development of wearable diagnostic devices for dynamic stress monitoring.

## Design of the vertically aligned electrode stack

The biosensor is comprised of MoS_2_ nanosheets embedded in the nanopores of a polyamide membrane through physiochemical absorption. Figure 1c shows a schematic representation of the electrode system and embedded MoS_2_. In contrast to more traditional thin-film electrode systems where electrodes are situated on the same planar axis, this work leverages both sides of the porous polyamide membrane to vertically align two palladium electrodes, enclosing the dispersed nanosheets. MoS_2_ nanosheets’ high surface-to-volume ratio and layered structure can contain a large capacity of antibodies, which reduce the sample volume to detect target molecules. The fabrication process details are described in the *Sensor Fabrication* sub-section of the Methods section. Our group has previously demonstrated detection of cortisol on a flexible substrate system^30^. However, in this work, with the orientation of the electrodes with respect to the dispersed nanosheets, sensing using EIS can be conducted through the membrane in a 3-dimensional (3D) mode, vis-a-vis the transverse or lateral mode used in the previous work. This vertically aligned electrode stack adds the benefits of capturing the electrochemical response through the entire volume of MoS_2_-dispersed sensing region between the electrodes, and allows for better isolation of the sensing region from environmental factors. These benefits enhance measured signal responses with low volumes of sweat and reduce non-specific responses^32–34^. This configuration also allows for the affinity-based assay to be constructed away from the physical electrode onto the MoS_2_, removing previous constraints on electrode material when considering crosslinker binding. Palladium was selected as an electrode material as it demonstrates low cost and stable electrochemical properties, but the method of MoS_2_ implementation allows for potentially any compatible electrode material to be used^35,36^. Dithiobis [succinimidyl propionate] (DSP) crosslinker was immobilized to the defects in the basal surface of the dispersed MoS_2_ nanosheets. Cortisol antibody was then bound to the DSP crosslinker, completing sensor functionalization. Cortisol in sweat can then bind freely to the immobilized antibody, modulating the electrochemical response. Figure 1c shows a schematic representation of the described assay construction for the sensor depicted in Fig. 1a (inset). The working electrode was designed in an open-face manner to allow for introduction of DSP, antibody, and doses.

## Transduction element (MoS_2_ nanosheets)

The prepared MoS_2_ nanosheets were deposited on the porous polyamide substrate to characterize their properties as they diffuse through the membrane. Scanning Electron Microscopy (SEM) was used to measure the size of the 2D nanosheets shown in Fig. 1b. The MoS_2_ flakes were observed inside the porous polyamide membrane and their size varied from around one hundred to a few hundred nanometers wide. Figure 1d shows the EDAX spectra related to the highlighted zone in the SEM micrograph. The observed carbon and oxygen peaks were due to the elemental composition of polyamide as a function of its hydrocarbon side chains. The broad peak at an energy level around 2.30 keV corresponds to the L-shell of Molybdenum (2.29 eV) and K-shell of Sulphur (2.31 eV) thereby indicating the conformal deposition of MoS_2_ nanosheets on porous polyamide.

**Figure 2:**
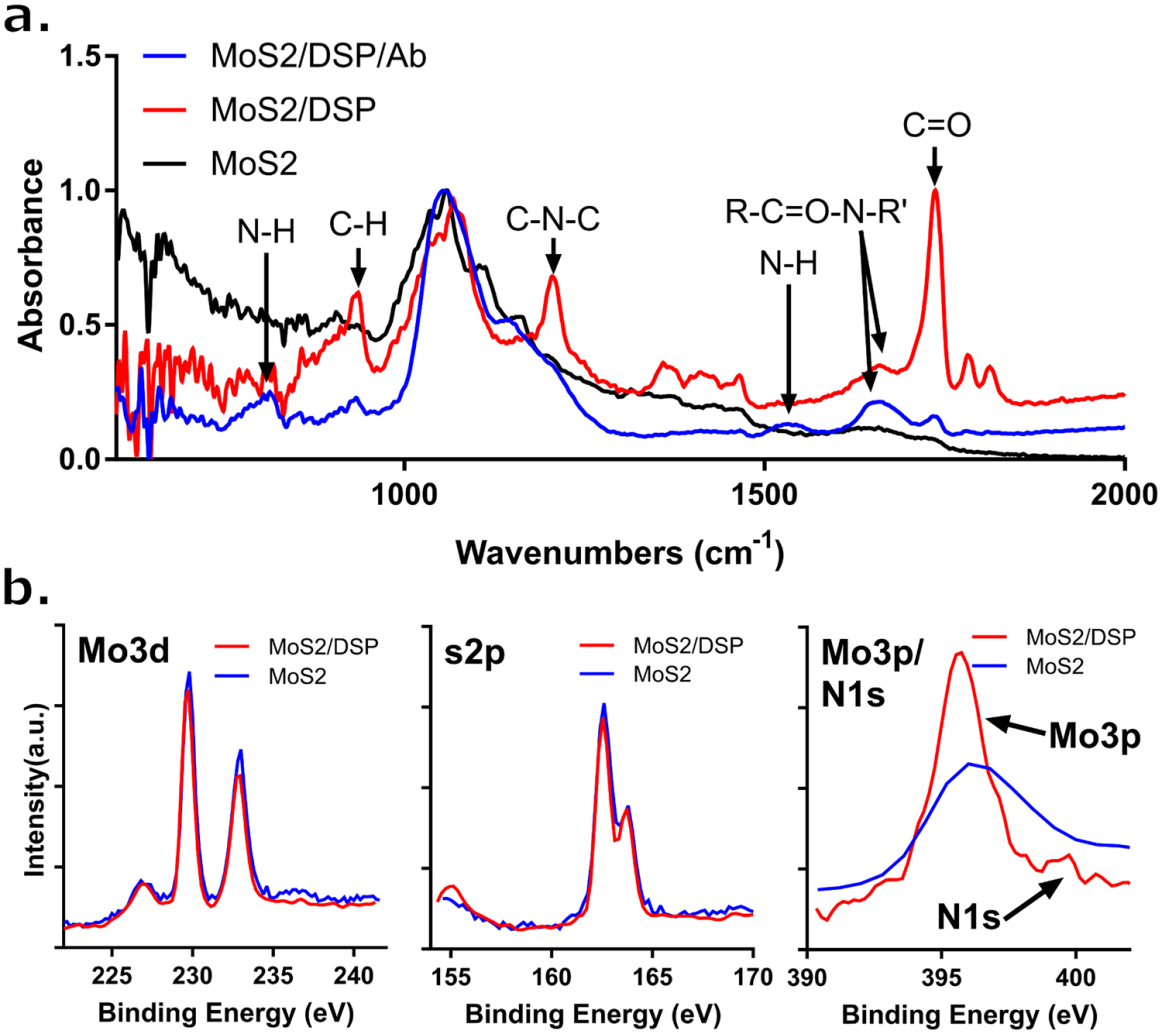
(**a**) ATR-FTIR spectra for the construction of the cortisol assay showing: (Black) MoS_2_, (Red) MoS_2_ + DSP, and (Blue) MoS_2_ + DSP+Antibody (**b**) XPS spectra showing Mo3d, S2p, and Mo3p/N1s core level peaks of MoS_2_ nanosheets (Blue) before and (Red) after DSP mobilization.

Figure 2a and Table 2 show further characterization performed using ATR-Fourier Transform Infrared Spectroscopy (ATR-FTIR) to confirm the subsequent binding of DSP and cortisol antibody during the sensor functionalization steps on the immobilized MoS_2_ nanosheets^37^. Post-DSP incubation, the appearance of three IR bands corresponding to C=O stretching modes (at 1813, 1783 and 1737 cm^−1^) indicate the formation of the DSP monolayer. The IR band for C-N-C stretch of the NHS group is also seen at 1205 cm^−1^. Second, after cortisol antibody incubation, the 1205 cm^−1^ peak disappear, indicating the detachment of the succinimidyl group (NHS) from the DSP layer. The N-H bending peak at ~1533 cm^−1^ and N-H out-of-plane bend at ~ 800 cm^−1^ appear after conjugation with the cortisol antibody, suggesting that the cortisol antibody was successfully conjugated to the DSP. This confirms that the DSP and cortisol antibody are bound to the MoS_2_ nanosheet surface.

**Table 2:** ATR-FTIR peak identification and assignment for assay functionalization steps.

Mode assignment	Description	Peak Position (cm^−1^)		
		*MoS*_2_	*MoS*_2_ + *DSP*	*MoS*_2_ + *DSP* + *Cortisol antibody*
*N-H*	N-H out of plane bend	N/A	N/A	~800
*C-H*	C-H sp^2^ bend	N/A	935	933 (decrease)
*C-N-C*	C-N-C stretch of NHS	N/A	1205	N/A
*C*=*O*	Carbonyl stretch of NHS	N/A	1737	1736 (decrease)
*C*=*O*	Carbonyl stretch of NHS	N/A	1783	1781 (decrease)
*C*=*O*	Carbonyl stretch of NHS	N/A	1813	1809 (decrease)
*R-C*=*O-N-R’*	Amides	N/A	1659	1662 (increase)
*N-H*	N-H bend	N/A	N/A	1533

Further validation was performed using X-ray Photoelectron Spectroscopy (XPS) to investigate the chemical components before and after the DSP modification of the MoS_2_ surface (Fig. 2b). The peaks of Mo3d (3d^5/2^ 230 eV, 3d^3/2^ 233 eV) and S2s (227 eV) of the DSP functionalized MoS_2_ nanosheets are found at the same binding energy positions as the non-modified MoS_2_. This suggests that the stoichiometric structure compositions for the MoS_2_ stayed unchanged after DSP functionalization. The appearance of the N1s peak at ~399.7 eV after modification suggests DSP functionalization onto MoS_2_ surface through the sulfur group mediated binding.

**Figure 3:**
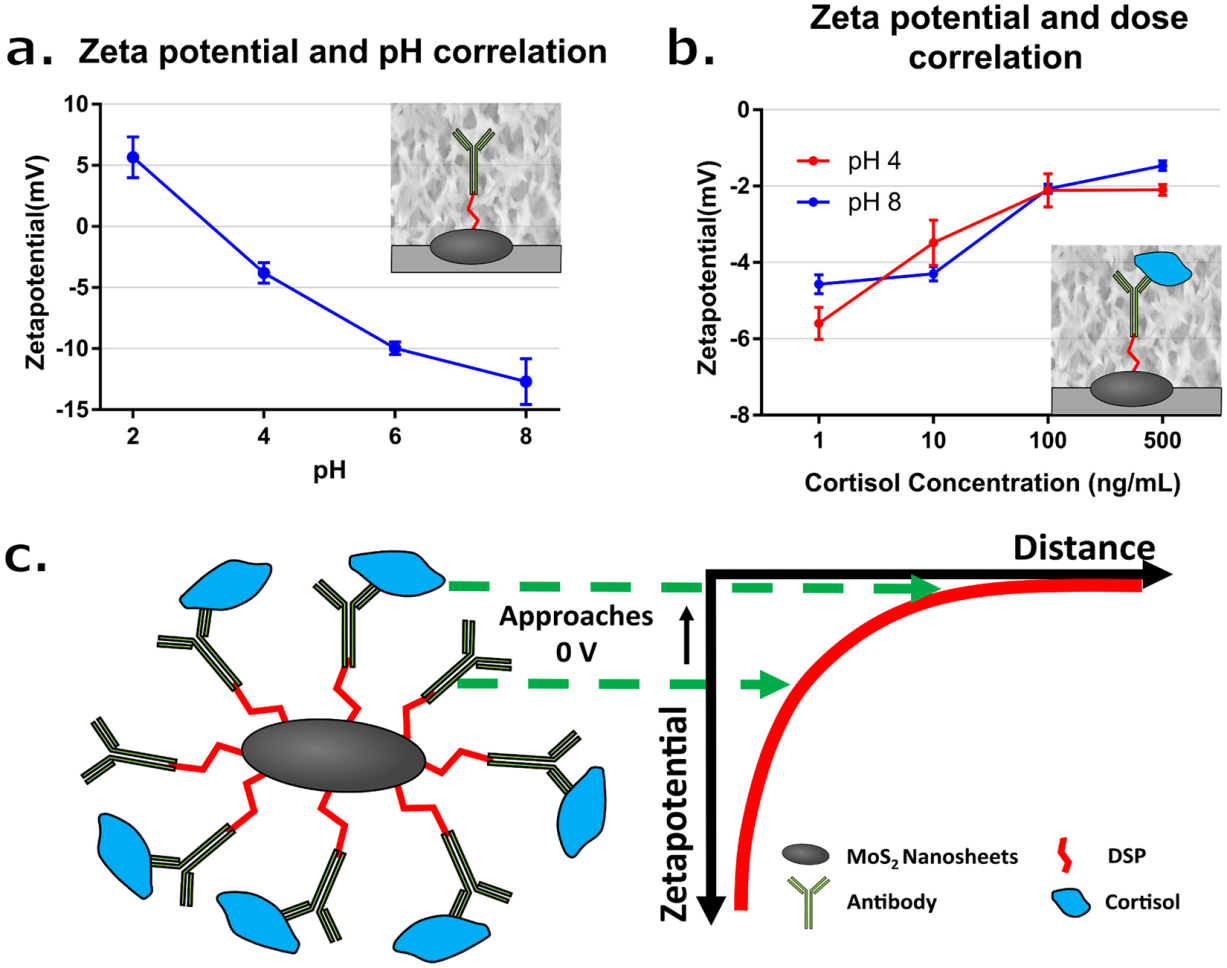
(**a**) Zeta potential of MoS_2_ nanosheet functionalized with cortisol antibody in pH values 2, 4, 6, and 8 of synthetic sweat (n = 3). (**b**) Zeta potential of MoS_2_ nanosheet functionalized with cortisol antibody with different concentrations of cortisol in (Red) pH 4 and (Blue) pH 8 synthetic sweat (n = 3). (**c**) Schematic drawing depicting the zeta potential difference after cortisol binding onto cortisol antibody.

Lastly, zeta potential measurement was used to confirm the binding of MoS_2_ nanosheets to the cortisol antigens (Fig. 3). The zeta potential, i.e. the electrostatic potential difference between an average point at the hydrodynamic shear plane and the bulk liquid, was measured representing the surface charge of the nanosheets. Figure 3a shows the zeta potential of MoS_2_ nanosheets functionalized with cortisol antibody in synthetic sweat at different values of pH. The value of zeta potential became less negative from pH 8 to pH 4 due to more H^+^ ions surrounding the MoS_2_ nanosheets. Substantial negative surface charges from pH 8 to pH 4 were observed. The typical pH range for the sweat of a normal healthy person is 4.5 to 7.0^38^. Figure 3b shows the zeta potential of MoS_2_ nanosheet functionalized with cortisol antibody incubated in different concentrations of cortisol in pH 4 and pH 8 synthetic sweat. Once cortisol molecule binds to antibody, the surface charge becomes less negative (Fig. 3c). These results suggest that the cortisol antigens have bound to the cortisol antibodies immobilized onto the MoS_2_ nanosheet surface and show a dose dependent electrochemical response across the pH range typical of human sweat. The functionalization is based on crosslinker immobilized to the defects in the basal surface of the chemically exfoliated MoS_2_ nanosheets. The results of the multiple techniques establish successful functionalization of the MoS_2_ in a range of conditions. These results suggest the assay will translate into good sensor performance and consistent response stability over a range of cortisol concentrations and across a wide range of sample pH.

## Calibration dose response of cortisol and continuous dosing

**Figure 4:**
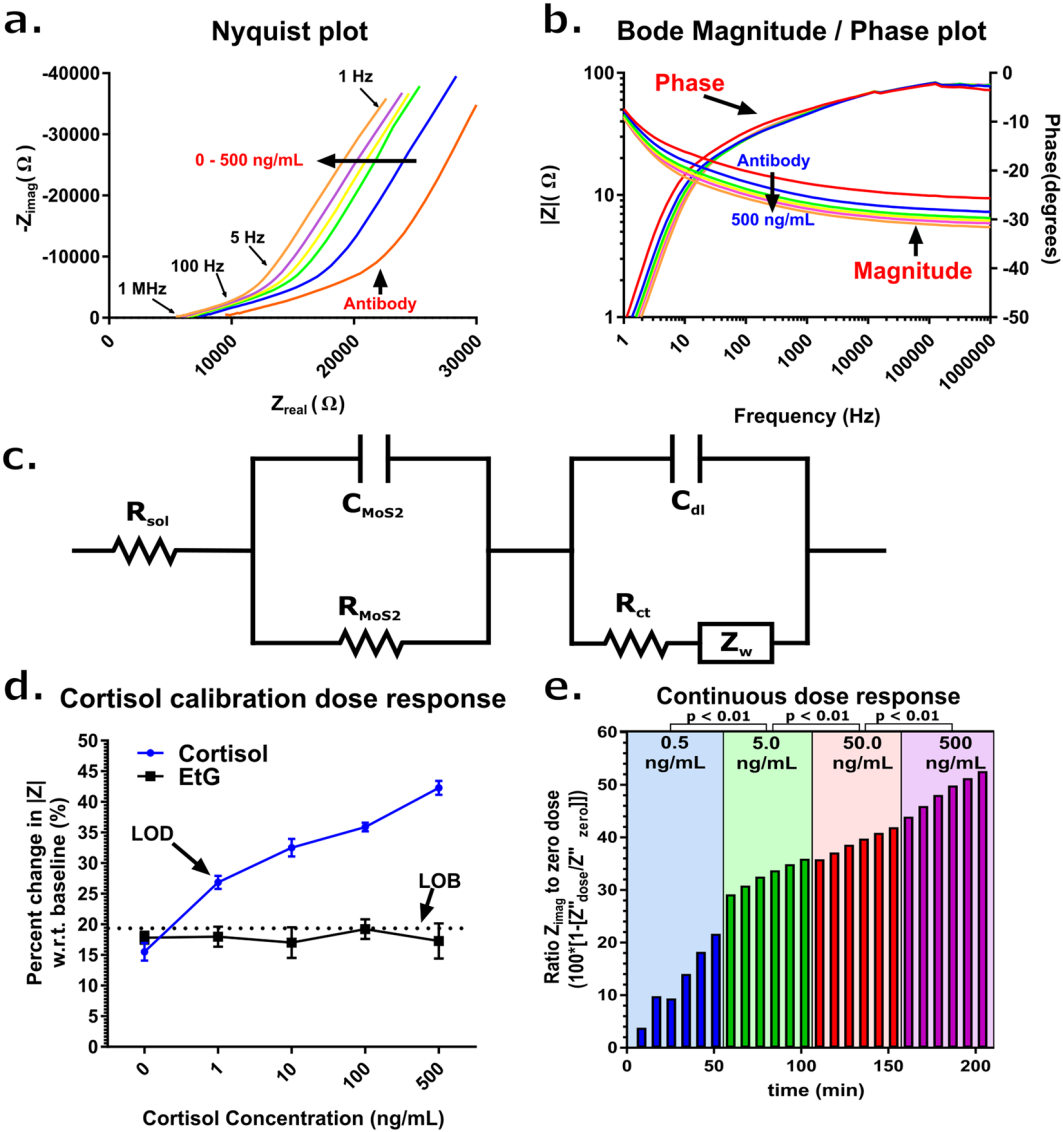
(**a**) Nyquist plot for the cortisol dose response. (**b**) Bode Magnitude and Phase plot for cortisol dose response (**c**) Modified Randles circuit representing the MoS_2_ functionalized electrode system comprised of passive electrical components. (**d**) (blue) Cortisol calibration dose response showing percent change in impedance with respect to the post-antibody baseline measurement for 0, 1, 10, 100, and 500 ng/mL of cortisol in human sweat (n = 3). (black) EtG cross-reactivity study (n = 3). (Dotted black) Limit of Blank. Error bars are Standard Error of Mean (**e**) 3-hour continuous detection of cortisol in human sweat with dose ranges: 0.5, 5.0, 50.0, 500.0 ng/mL. Impedance measurements are ratio of Z_imag_ measurement for given dose point to a 0-dose baseline. p-values depicted are the comparison of the statistical significance between the measurements in each respective concentration range.

After sensor functionalization, samples of human sweat, spiked with 0, 1, 10, 100, and 500 ng/mL of cortisol, were applied to the sensing region of the sensor. The Nyquist plot in Fig. 4a shows how the EIS response changed with respect to increasing dose of cortisol. The plot is divided into regions that are affected by different portions of the equivalent Randle’s circuit for the sensor represented in Fig. 4c. The first region (<5 Hz) represents the Warburg impedance of our equivalent circuit. This component, which is created by the diffusion of ions from the human sweat buffers at low frequencies. The second region (5–1000 Hz) represents the capacitance of the EDL^39^. As cortisol dose concentration increases, the formation of the cortisol-antibody complex within the EDL increases the capacitive reactance (C_dl_ and C_MoS2_) and causes the observed shift in the Nyquist plot in Fig. 4a. The change of R_MoS2_ is due to the properties of the MoS_2_ nanosheets. This decrease in R_MoS2_ is attributable to the accumulation of charge causing the semiconducting nanosheets to become more conductive. This distinguishable feature is primarily a function of the semiconducting MoS_2_ nanosheets themselves and represents a means by which to measure cortisol binding in the constructed affinity assay^40^. As a result, sensing is accomplished through changes in capacitive reactance of the system so that a dose dependent shift in phase may not be apparent. Though, a greater phase shift can be seen at a lower (<10 Hz) frequency, diffusion-driven mechanisms start to dominate in this regime, so a higher frequency is desired for analysis. With this in mind, analysis was conducted at 100 Hz. Theoretical fits of the experimental results for the capacitive and resistive components are summarized in Table 3, which was constructed by using Z-view software to fit the modified Randle’s circuit to the experimental data, and shows an increase in EDL capacitance (C_dl_, C_MoS2_) as dosage concentration increases with a 336% and 58% increase respectively. In addition, the R_MoS2_ also decreases (18%) as a function of dosage concentration. These two components of the equivalent circuit dominate the dose response and indicate that the sensor is sensing capacitive reactance changes created by cortisol binding to the MoS_2_ nanosheets.

**Table 3:** Theoretical fit of experimental results for the capacitive and resistive electrical components of the Modified Randles Circuit.

Cortisol Concentration (ng/mL)	Components of Modified Randles Circuit				
	Rsol (kΩ)	Rct (μΩ)	Cdl (nF)	RMoS2 (kΩ)	CMoS2 (μF)
1	6.5	2.4	189.4	10.4	2.4
10	6.2	2.9	260.9	9.5	2.9
100	5.8	3.6	550.0	9.3	3.6
500	5.7	3.8	824.1	8.8	3.8

The cortisol dose response is also represented as Bode phase and magnitude plots in Fig. 4b. |Z| reflects the total impedance of the system and can be used as a measure of both capacitive and resistance changes. Figure 4b shows that from the baseline measurement, there is a progressive decrease in impedance with respect to increasing cortisol concentration. The calibration dose response of the sensor shown in Fig. 4c is represented as a percent change in |Z| impedance from the baseline measurement post-antibody functionalization at 100 Hz. There is a consistent decrease in impedance as the dosage concentration of cortisol increases. The percent change with respect to a post-functionalization baseline measurement was observed to be: 15.5% +/− 1.5% for 0 ng/mL, 26.85% +/− 1.1% for 1 ng/mL, 32.5% +/− 1.5% for 10 ng/mL, 35.9% +/− 0.7% for 100 ng/mL, and 42.3% +/− 1.1% for 500 ng/mL. Additionally, in order to establish that the observed signal response was specific to the cortisol binding events occurring on the MoS_2_ nanosheets and not due to non-specific and physically absorbed mechanisms occurring on the polyamide substrate, a control study with the absence of DSP crosslinker was conducted. The results of this study are included in Supplementary Figure S1. The protocol followed was identical to the one performed for the calibration dose response, but the DSP crosslinker used to bind the antibody to the MoS_2_ nanosheets was excluded. This left no place for the antibody to chemically bind upon the MoS_2_ nanosheets. The observed signal from applied cortisol doses due to non-specific interactions ranged from −0.6% +/− 1.7% to 5.5% +/− 2.1% in the absence of DSP. This is significantly less than the 15.5% +/− 1.5% to 42.3% +/− 1.1% change in impedance observed with DSP included in the protocol, thus establishing the specificity of the measured impedance response and its association with cortisol binding to the MoS_2_ nanosheets.

Human sweat contains a variety of solutes and interferents that are capable of interfering with the detection of EDL binding, thus increasing the noise present in the measurement. An additional control experiment was conducted to characterize our sensor’s sensitivity to non-specific biomarkers. Here, ethyl glucuronide (EtG) was used as a cross-reactive molecule to probe the specificity of the system. EtG is expressed when ethanol is broken down in the liver, but exists at basal levels in similar concentrations to the physiological range of cortisol in human sweat, so it poses as a good cross-reactive molecule for this study. The same protocol was followed as for cortisol dose response, but with dose concentrations of EtG while maintaining the cortisol specific antibody. The response seen in Fig. 4d shows impedance changes that are within the noise threshold of the system for all concentrations of EtG. Hence, the sensor is not sensitive to non-specific adsorption associated with EtG. This again validates that the change in impedance is a function of cortisol specifically binding to functionalized MoS_2_. Therefore, with a Limit of Blank (LOB) calculated as 19.65%, a Limit of Detection (LOD) of 1 ng/mL within human sweat can be established, indicating that the sensor can detect cortisol in biological samples across physiologically relevant concentrations between 1 and 500 ng/mL.

Figure 4e depicts the continuous response of the sensor for four cortisol concentration regimes (0.5, 5, 50, and 500 ng/mL). In each regime, six doses of a single cortisol concentration were applied at a rate of 3 μL every 7 minutes to the sensor. This protocol was adopted from a modified protocol previously reported by our group^41^. For this study, Z_imag_ at 100 Hz was used to report the ratio changes in impedance due to the dominant capacitive reactance effects, as reported in Table 3. As dose concentration increased logarithmically, it was observed that the change in impedance responded incrementally to the increasing doses of cortisol. An unpaired t-test with a 95% confidence interval was performed comparing each range of applied dose concentration to one another. A p-value less than 0.05 was observed for every comparison of dose ranges. This showed that the dose ranges were statistically significant from one another and were differentiated during continuous measurement. With a total duration of 215 minutes, the sensor showed minimal signs of saturation. This indicates that the sensor is responsive to concentrations of cortisol over a functional period of over 3 hours and therefore is suitable for continuous sensing at both high levels and low levels of cortisol from low volumes of human sweat.

## Wearable form factor cortisol sensor device testing

**Figure 5:**
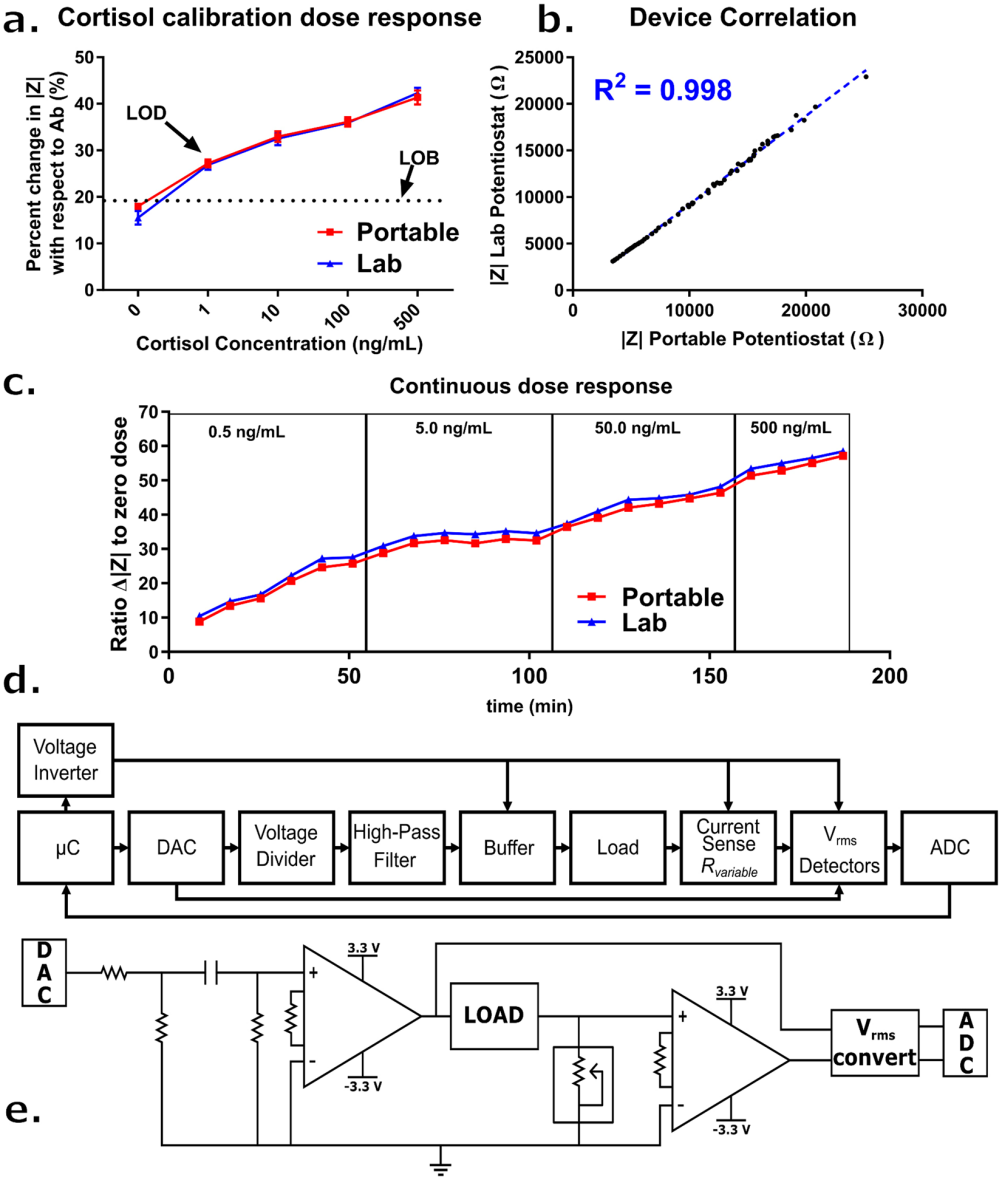
(**a**) (Red) Portable and (Blue) Lab potentiostat comparison for the cortisol calibration dose response showing percent change in |Z| with respect to the post-antibody baseline measurement for: 0, 1, 10, 100, and 500 ng/mL of cortisol in human sweat (n = 3). Error bars are standard error of mean. (**b**) Portable and Lab Potentiostat impedance measurement correlation for all raw measurements represented in continuous and calibration dose response data set. (**c**) (Red) Portable and (Blue) Lab Potentiostat comparison of |Z| response for the 3 hour continuous detection of cortisol in human sweat with dose ranges: 0.5, 5.0, 50.0, 500.0 ng/mL. (**d**) Block diagram of the portable potentiostat circuit implementation. (**e**) Circuit drawing of the portable potentiostat.

A portable electronic device, capable of performing impedance measurements at 100 Hz was developed to demonstrate proof of feasibility and translatability of the proposed cortisol biosensor into an economical and compact form factor towards eventual wearable integration. The efficacy of the device was established by simultaneously performing measurements for the calibration dose response and continuous dosing studies on both the lab potentiostat and developed electronics. Figure 5d shows a block diagram representation of the circuit, while Fig. 5e shows the circuit schematically. For this work, the goal was to develop a research prototype capable of reporting values of |Z| to the same degree of accuracy on the cortisol sensor when compared to the benchtop instrument. All electronic data was reported as |Z| for this study. Details on the construction and testing of the device are included in the *Portable Potentiostat* sub-section of the Methods.

### Rapid detection of cortisol

Based on the results collected on the lab potentiostat presented in Fig. 4, a 100 Hz single frequency EIS measurement was used for the quantification of cortisol on the developed portable electronics platform. The device takes 8–10 seconds per single frequency measurement in contrast to a full frequency sweep (0.1 Hz–100 MHz) that takes approximately three minutes. Thus, single frequency analysis allows for rapid detection of cortisol which in turn allows for reduction of the portable device footprint as well as the power consumption of the device, both of which are critical considerations for wearable applications.

### Performance comparison of Wearable form factor device

Figure 5a shows the comparison of the cortisol calibration dose responses for the lab potentiostat and the device, both demonstrating the same LOD of 1 ng/mL and detection range of 1–500 ng/mL. An unpaired t-test with a 95% confidence interval was performed on the three replicates of the two devices to establish the performance of the portable device. Each comparison of dose concentration yielded a p-value > 0.65, demonstrating that there is no statistical significance in the resulting percent changes and giving a quantifiable measure to the device’s comparable performance to the lab potentiostat.

Figure 5c compares the continuous dosing response of both devices. Despite a small offset in calculated impedance, which is consistent and predictable across the study, the reported value from the electronic device is an accurate measure of the impedance reported by the benchtop instrument. When the continuous responses of the two devices are plotted against each other, a slope of 0.999 with an R^2^ value of 0.999 is realized. This demonstrates that the device can distinguish even minute changes in impedance to the same degree as the lab potentiostat for this application space. Similarly, when all raw |Z| measurements from both the dose response and continuous studies were plotted against each other, an R^2^ value of 0.998 was observed (Fig. 5b), showing that the device maps linearly with the lab potentiostat and reports comparable values of impedance.

### Device charge and battery life

The device consumes less than 4 μA_rms_ during its <10 second measurement highlighting the ultra-low current consumption characteristics of the electrochemical transduction method. With a measurement refresh rate of 7-minutes the device can remain idle for ~98% of the time allowing for future iterations of the device to have an exceptional charge lifetime.

### Flexibility and wearability

**Figure 6:**
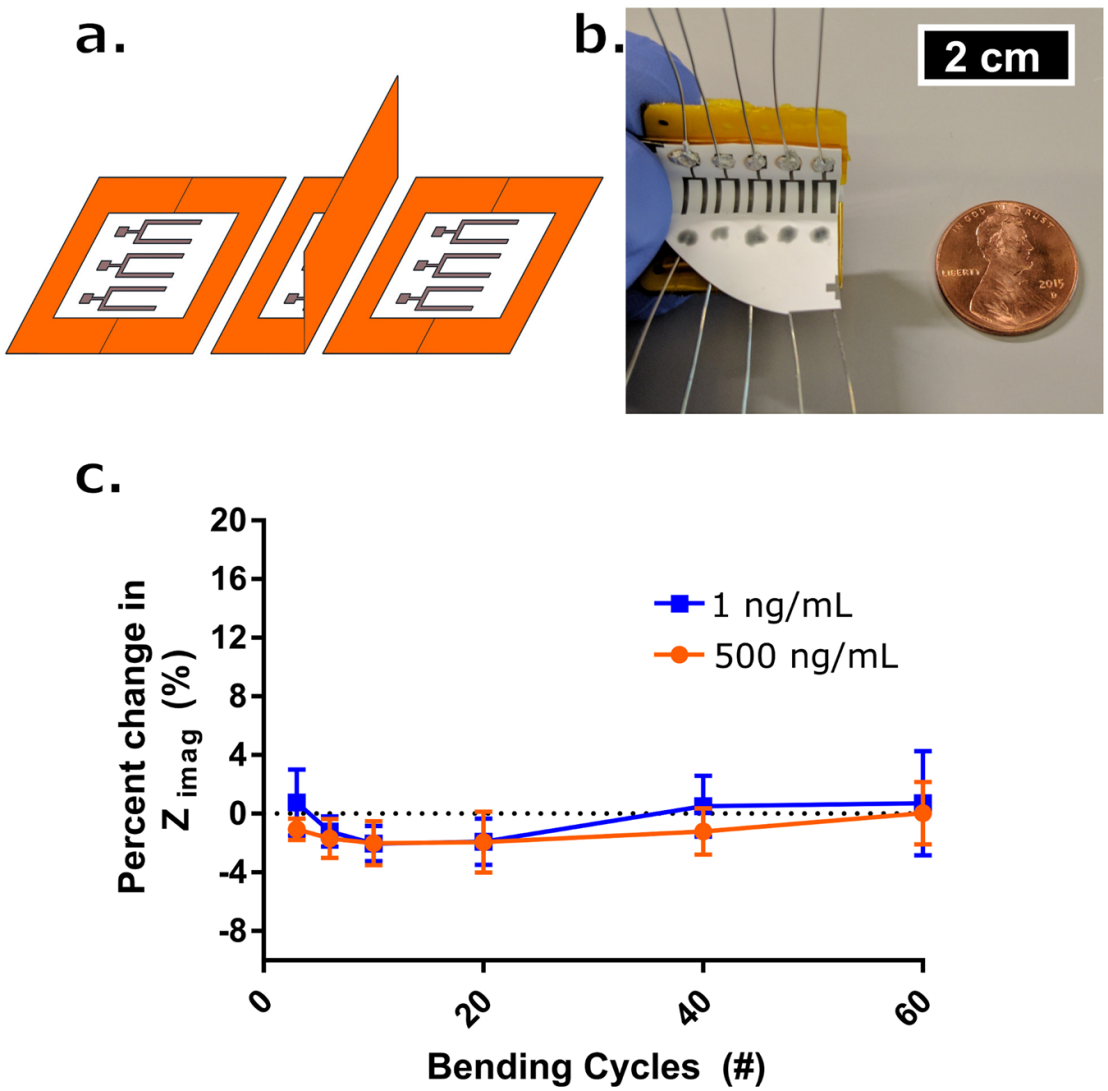
(**a**) Schematic drawing for one complete bending cycle of the sensor. One cycle is comprised of (left) unbent state, (middle) 90° flexion motion, and (right) return to unbent state at which point a measurement occurs. (**b**) Picture of bending apparatus with sensor array affixed. Penny for reference. (**c**) Percent change in Z_imag_ impedance with respect to the initial measurement post-cortisol dosing and 7 minute incubation time (blue box – 1 ng/mL, red circle – 500 ng/mL) after # of bending cycles (n = 3). Error bars are standard error of mean.

A bending study was conducted to demonstrate the sensor’s resilience to physical deformation. The purpose of the study was to demonstrate that cyclic bending cycles would not degrade the cortisol affinity assay constructed upon the MoS_2_ nanosheets. This was not meant to be an exhaustive study of the impact of physical stressors on sensor performance, but rather establish the kind of impedance response that can be expected during cyclic stress. The protocol followed was adapted from a similar bending study reported previously by our group^39^. An array of sensors was interfaced with a Kapton passivated hinge as shown in Fig. 6b. A single bending cycle in this study was defined as the sensor undergoing 90 degrees flexion motion, and returning to an unbent state (Fig. 6a). Using equation 1, this correlates to a ~1% bending compression experienced at the sensing region per cycle for a 5 mm radius of curvature, and 110 µm membrane thickness. The protocol followed for this study is outlined in “*Affinity assay functionalization and protocols”* of the Methods. Experiments were conducted on the lab instrument.

Figure 6c shows the percent change in impedance after multiple bending cycles with respect to a measurement taken on sensors incubated with cortisol but before any bending cycles had been performed. The upper and lower extremes of cortisol concentrations were investigated (1 and 500 ng/mL). The graph shows that there is a minimal change in impedance (~−2–1%) for both concentrations even after 60 bending cycles. This establishes that the reported impedance values recorded before any bending cycles post-introduction of a given cortisol dose were preserved after consecutive loads of stress. This demonstrates that the bending had no significant impact on the sensor’s ability to accurately report the impedance value for a given cortisol dose, and that the assay maintained its stability.

1 $${\varepsilon }_{electrode}=\frac{membrane\,thickness}{2\ast radius\,of\,curvature}$$

## Conclusion

In this manuscript, detection of cortisol in physiologically relevant ranges of perspired human sweat was demonstrated at low volume (1–5 μL) using a vertically-aligned metal electrode sensor system in conjunction with confined semi-conductive MoS_2_ nanosheets. The sensor’s specificity to cortisol was achieved through an affinity assay using MoS_2_ nanosheets functionalized with cortisol antibody as the active sensing element. Sensing was achieved by measuring impedance changes associated with cortisol binding at the MoS_2_ nanosheet interface using electrochemical impedance spectroscopy. These changes were determined to be primarily a result of capacitive reactance due to the modulation of the surface charge along the MoS_2_-double layer interface. Continuous dosing studies demonstrated the sensor’s ability to discriminate between four cortisol concentration ranges (0.5, 5, 50, 500 ng/mL) for a 3 + hour duration. The construction of the affinity assay upon MoS_2_ was validated using multiple surface characterization techniques as well as an impedance study in the absence of the DSP crosslinker. Sensor specificity was established through a cross-reactivity study using ethyl glucuronide. The impact of mechanical stress was studied by evaluating the impedance response to cyclic bending, demonstrating a minimal loss in signal with respect to applied stress. This work demonstrates the first example of a portable perspired sweat cortisol monitor through non-faradaic impedance spectroscopy, demonstrating comparable detection of cortisol to a laboratory potentiostat. This work has the potential to vastly improve the health monitoring abilities of both sedentary populations, where the amount of perspired sweat is minimal, as well as in specialized applications such as space travel, where portable non-invasive sensing can provide valuable diagnostic feedback to high risk users. Further study of this research prototype biosensor will be needed to establish the robustness and reliability necessary for commercial implementation.

## Methods

### Materials and Reagents

0.2 μm pore size polyamide substrates were purchased from GE Healthcare Life Sciences (Piscataway, NJ, USA). Dithiobis [Succinimidyl Propionate] (DSP), its solvent Dimethyl Sulfoxide (DMSO), and 150 mM Phosphate Buffered Saline (PBS) were ordered from Thermo Fisher Scientific Inc. (Waltham, MA, USA). The α-cortisol antibody and cortisol hormone (Hydrocortisone) were ordered from Abcam (Cambridge, MA, USA). Single donor human sweat was purchased from Lee biosolutions Inc. (St. Louis, MO, USA). Molybdenum Disulfide (MoS_2_) Crystal was purchased from SPI supplies (West Chester, PA, USA) and was diluted in Milli-Q deionized water (18 MΩ).

### MoS_2_ preparation

MoS_2_ nanosheets were synthesized from bulk MoS_2_ using a modification of the chemical exfoliation process described by Zhou *et al*.’s work^42^. Bulk MoS_2_ of 5.0 mg was mixed in 5 ml of 45%/55% Ethanol/DI water solution and was sonicated for six hours. The exfoliated MoS_2_ nanosheets were centrifuged at 3000 rpm for 20 min to isolate nanosheets of one to few layers in the supernatant, leaving un-exfoliated bulk in a pellet at the bottom of the tube. 70% of supernatant by volume was transferred into a separate tube and heated at 70 °C for one hour, evaporating remaining ethanol. DI water was added post-evaporation to regain original volume to maintain constant concentration of MoS_2_ across preparations.

### Sensor fabrication

100 nm palladium working and reference electrodes of the MoS_2_ sensors were fabricated upon the polyamide membranes in a two-step thin film cryo-evaporation deposition process using in-house fabricated shadow mask stencils. Stencils were fabricated using a Kapton sheet base substrate. Electrode geometries were designed in AutoDesk^TM^ AutoCAD and cut into the Kapton substrates using a Spectra-Physics Spirit laser system. Following the first step of the deposition process, 10 µL of 1 mg/mL MoS_2_ solution in Milli-Q deionized water was dispersed through vacuum assistance over the sensing region of the electrode, on the non-electrode deposited side of the membrane. Following the dispersion of MoS_2_, the second electrode pattern was deposited completing the fabrication processes. The second shadow mask was aligned to the first deposited electrode using alignment marks built into the mask design files and a backlit alignment platform. Wires, used to interface with the lab and portable instruments, were connected to the contact pads of the sensors through use of silver conductive epoxy. The calibration dosing response and continuous dosing experiments were conducted using individually diced sensors. For the bending study alone, sensors were arrayed for testing to ensure that the bending stress administered to each sensor was equal amongst all replicates.

### Affinity assay functionalization and protocols

All experiments were conducted on benchtop, with samples applied between the two arms of the working electrode with a micropipette. For all experiments other than the bending study, sensors were individually diced for testing. After sensor fabrication, 5 μL of 10 mM DSP in dimethyl-sulfoxide (DMSO) was incubated for three hours in dark. After a 5 μL PBS wash to remove any unbound DSP crosslinker, 5 μL of 100 ug/mL alpha-cortisol antibody was applied and incubated for 15 minutes followed by another 5 μL PBS wash step. A zero-dose measurement was first taken with cortisol-free human sweat. For the calibration dose response 5 μL of cortisol doses (1, 10, 500, 1000 ng/mL) prepared in human sweat were added to the sensing region and allowed to incubate for 7 minutes before a measurement was taken. Subsequent doses were added from lowest concentration to highest, and were all allowed to incubate for the same 7 minutes before measurements were taken. The described protocol is adapted from our group’s previously published ZnO cortisol biosensor work, which was performed on the same polyamide substrate^30^. Volumes for the functionalization steps were maintained at or below 5 μL due to the effective volume of the sensor. The polyamide membrane is 110–150 μm in thickness. The sensing region of the sensor is 3 mm × 6 mm in area allowing for ~2.5 μL of total volume to saturate the sensing region. 5 μL volume selection allowed for complete saturation of the sensing region making it appropriate for functionalization and wash steps. For the continuous dosing experiment, doses of 0.5, 5.0, 50 and 500 ng/mL of cortisol in sweat were added to the sensing region progressively. Six applications of the same dose concentration were added at a rate of 3 μL per 7 minutes before moving to the subsequent concentration, this continued through 500 ng/mL for a total duration of ~215 minutes. For the calibration dose response and continuous sensing simultaneous measurements were performed on a Gamry Reference 600 Potentiostat (Gamry Instruments, Warminster, PA, USA), and the developed portable potentiostat. For the cross-reactivity study, the protocol remained identical, but concentrations of cortisol were replaced with ethyl glucuronide (EtG) also in human sweat. For the bending study, arrayed sensors were affixed to a Kapton passivated hinge before any functionalization steps. The protocol for the calibration dose response was followed for the bending study, but rather than administering logarithmically increasing concentrations of cortisol a single dose (either 1 ng/mL or 500 ng/mL) was applied to the sensing region, and measurements were conducted after a number of bending cycles. Bending cycles consisted of a 90° flexion motion and return to an unbent state. All measurements were conducted in unbent state. All studies were conducted in triplicate with exception of the continuous study.

### Infrared spectroscopy (ATR-FTIR)

Infrared spectra of MoS_2_ nanosheets and functionalized MoS_2_ nanosheets were recorded with a Thermo Scientific Nicole iS50 FT-IR using an Attenuated Total Reflectance (ATR) stage. The tool was equipped with a deuterated triglycine sulfate (DTGS) detector and KBr window. A Harrick VariGATR sampling stage with a 65° Germanium ATR crystal was used in this study. ATR-FTIR specimens were prepared by drop casting the solutions on silicon wafer with 300 nm SiO_2_ as substrate. The contact area was about 1 cm^2^. All spectra were recorded between 4000 and 600 cm^−1^ with a resolution of 4 cm^−1^ and 256 scans.

### X-Ray Photoelectron Spectroscopy (XPS)

XPS spectra were acquired to detect presence of DSP on MoS_2_ conjugates using a PHI 5000 Versa Probe II with a monochromatic Al Kα radiation (hν = 1486.6 eV). All evaluations were taken at a 45° takeoff angle with respect to the sample surface. Spectra were obtained with a 0.2 eV step size and 23.50 eV pass energy. The base pressure in the analysis was 1.6 × 10^−8^ Torr. Samples were prepared using silicon chips with 300 nm SiO_2_ as substrate. Repeated drop casting of MoS_2_ conjugates were made to build up film thickness. Samples were dried in a vacuum chamber. All binding energies were corrected for the charge shift using the C 1 s peak of graphitic carbon (BE = 284.8 eV) as a reference.

### Zeta Potential and Size Measurements

Zeta Potential and Size of MoS_2_ suspended in synthetic sweat were measured using Malvern ZetaSizer Nano ZS (Malvern Instruments). 10 μL of MoS_2_ nanosheets, 10 μL of DSP in dimethyl-sulfoxide (DMSO) and 10 μL of alpha-cortisol antibody was incubated for three hours. After three hours, 970 μL of different pH synthetic sweat was added and triplicate measurements were taken for each dilution. Zeta Potential was computed from the measured electrophoretic mobility using the relationship:

2 $$\mu =\frac{\varepsilon .\,{\rm{\zeta }}}{\eta }$$

where ε is the dielectric constant, and η is dynamic viscosity of the dispersion medium, and ζ is Zeta Potential.

### Portable Potentiostat

A schematic representation of the portable potentiostat circuit and block diagram detailing the major aspects of the device are shown in Fig. 5d and e respectively. The device used in this work is a research prototype to demonstrate translatability to portable and eventually wearable form factors. The device is controlled using an Arduino Uno R3 microcontroller unit, which was used to power the individual components as well as to acquire and report. A TL7660 voltage converter was used to create a negative voltage rail for the rail-to-rail amplifiers, as the microcontroller cannot inherently handle the negative voltages needed for this biosensing application. A MCP4725 Digital-to-Analog converter was used to generate the 100 Hz sine wave used as an excitation signal. The signal was conditioned using a voltage divider and high pass filter to remove any DC-offset and reduce the amplitude of the excitation signal creating a 20 mV_rms_ signal appropriate for electrochemical measurements. The conditioned signal was fed into a buffer circuit (INA 121 Instrumentation Amplifier) before interfacing with the epoxied wires of the electrode. The current at the working electrode was monitored using a current sense circuit (INA 121 Instrumentation Amplifier). A digital potentiometer was used to configure the sense resistor to different current ranges depending on the impedance range of the sensor. The excitation signal and voltage output of the I-V converter were fed into a V_rms_ converter (AD 736) to generate a DC signal analogous to the gain difference in the two signals. The signals from the converter were reported back to the microcontroller using a 12-bit ADS1015 analog-to-digital converter and used to calculate the effective impedance of the system. All parameters were established using lab instrument data. For this research prototype, all analysis was conducted off-instrument. All signals were tested for accuracy using an Agilent Technologies DC Power Analyzer (Agilent Technologies, Santa Clara, CA).

## Electronic supplementary material

Supplementary information
