# Supplementary material for: Portable biosensor for monitoring cortisol in low-volume perspired human sweat
Source: Sci Rep. 2017 Oct 17;7:13312. doi: 10.1038/s41598-017-13684-7 (PMC5645384; doi:10.1038/s41598-017-13684-7)
Supplement: Supplementary file 1 — Supplementary information [file 41598_2017_13684_MOESM1_ESM.pdf]

Supplemental information for the article:

Portable biosensor for monitoring cortisol in low-volume perspired human sweat

David Kinnamon[a+], Ramesh Ghanta[a+], Kai-Chun Lin[a+], Sriram Muthukumar[b], Shalini Prasad[a*]

[a] Department of Bioengineering, University of Texas at Dallas, 800 West Campbell Road, Richardson, TX 75080, USA
[b] Enlisense LLC, 1813 Audubon Pond way, Allen, TX 75013, USA
[+]Denotes equal contribution of authorship
[*]Denotes corresponding author

Supplemental Data Entry (S1)

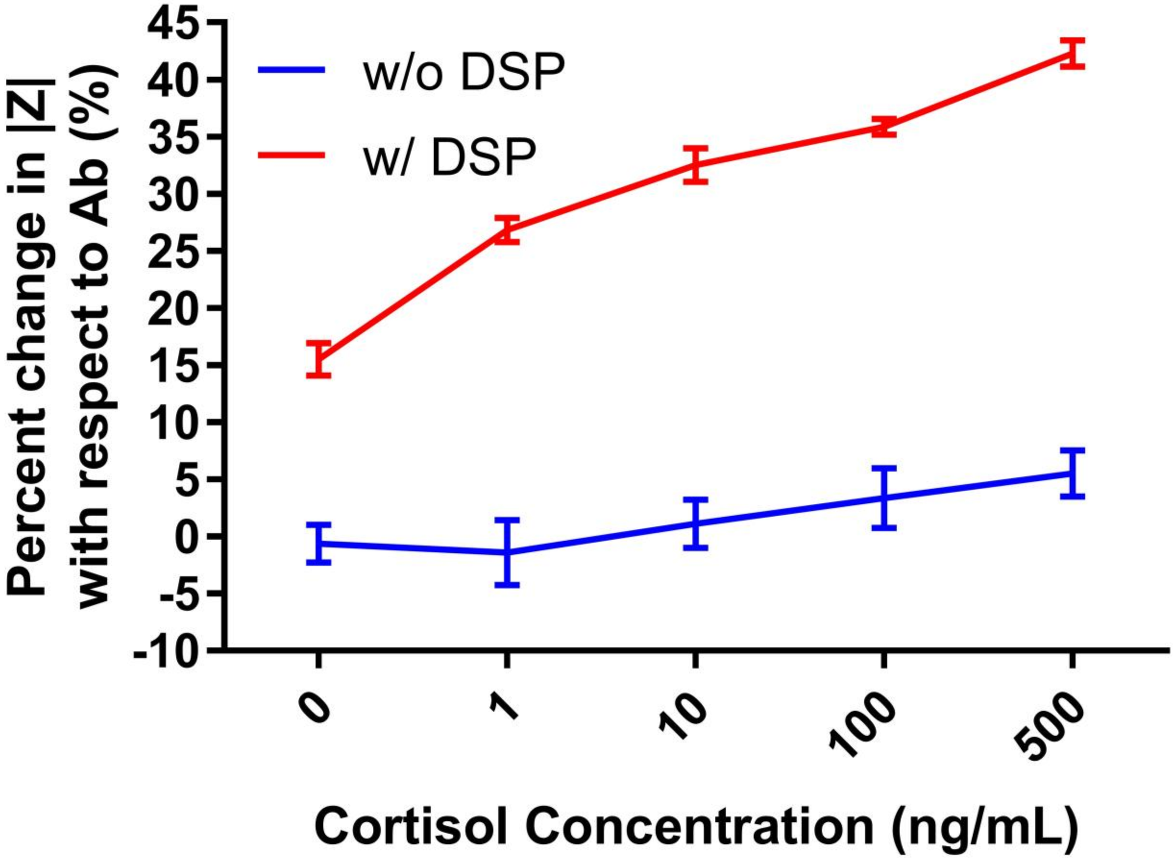

Figure S1 Comparison percent change in |Z| of the constructed cortisol affinity assay when DSP crosslinker is included in the protocol (red), and when it is not included (blue). (n = 3) replicates, represented error bars are SEM.

Figure S4 shows the results of a negative control study conducted to establish that the specific signal from the calibration dose response presented in the main text is from bound antibody to the MoS_2 nanosheets though the DSP crosslinker, and not a result of binding to physically absorbed antibody that could exist in the pores of the polyamide membrane. In this experiment, the CDR protocol detailed in the main text was followed, but sensors were incubated with blank DMSO without DSP for three hours, as opposed to the 10 mM of DSP in DMSO crosslinking solution. The results of the study show that there is only about a 5.5% +/- 2% change in impedance from the cortisol-free zero dose to the max concentration of 500 ng/mL when DSP is excluded from the protocol. This contrasts with the 27% +/- 1.1% increase seen from the cortisol-free zero dose to the max concentration of 500 ng/mL when DSP is included in the protocol. This shows that without the DSP crosslinker bound to the MoS_2 nanosheets the assay is significantly less sensitive to changes in cortisol concentration. This is because any cortisol that is binding to physically absorbed antibody off of the MoS_2 surface is not captured by the C_{MoS2}

and R_{MoS2} changes leveraged for sensing in this biosensor, and are only contributing to the small 5.5% change in impedance seen in the data set.
